# Chemical and Mechanical Defenses Vary among Maternal Lines and Leaf Ages in *Verbascum thapsus* L. (Scrophulariaceae) and Reduce Palatability to a Generalist Insect

**DOI:** 10.1371/journal.pone.0104889

**Published:** 2014-08-15

**Authors:** Christina Alba, M. Deane Bowers, Dana Blumenthal, Ruth A. Hufbauer

**Affiliations:** 1 Graduate Degree Program in Ecology and Department of Bioagricultural Sciences and Pest Management, Colorado State University, Fort Collins, Colorado, United States of America; 2 University of Colorado at Boulder, Department of Ecology and Evolutionary Biology, Boulder, Colorado, United States of America; 3 USDA-ARS, Fort Collins, Colorado, United States of America; Umeå Plant Science Centre, Umeå University, Sweden

## Abstract

Intra-specific variation in host-plant quality affects herbivore foraging decisions and, in turn, herbivore foraging decisions mediate plant fitness. In particular, variation in defenses against herbivores, both among and within plants, shapes herbivore behavior. If variation in defenses is genetically based, it can respond to natural selection by herbivores. We quantified intra-specific variation in iridoid glycosides, trichome length, and leaf strength in common mullein (*Verbascum thapsus* L, Scrophulariaceae) among maternal lines within a population and among leaves within plants, and related this variation to feeding preferences of a generalist herbivore, *Trichopulsia ni* Hübner. We found significant variation in all three defenses among maternal lines, with *T. ni* preferring plants with lower investment in chemical, but not mechanical, defense. Within plants, old leaves had lower levels of all defenses than young leaves, and were strongly preferred by *T. ni*. Caterpillars also preferred leaves with trichomes removed to leaves with trichomes intact. Differences among maternal lines indicate that phenotypic variation in defenses likely has a genetic basis. Furthermore, these results reveal that the feeding behaviors of *T. ni* map onto variation in plant defense in a predictable way. This work highlights the importance of variation in host-plant quality in driving interactions between plants and their herbivores.

## Introduction

Host-plant quality, which is the balance between nutrients and defenses in plant tissues that influences herbivores [1], varies within as well as between species. Intra-specific variation in host-plant quality can shape herbivore community dynamics by influencing herbivore abundance and distribution [2], [3], for example when herbivores preferentially feed on different plant genotypes within a population [2]. Further, because differences in nutritional value and levels of defense among individual plants or plant tissues affect patterns of herbivory, they may feed back to determine plant fitness [4]. Intra-specific variation in plant quality also influences higher trophic levels, due to both differences in herbivore abundance and differences in the quality of herbivores as prey or hosts [5], [6]. Thus, characterizing intra-specific variation in host-plant quality, which is often manifested among maternal lines within a population, lies at the foundation of understanding plant-herbivore and herbivore-enemy interactions.

Defenses against herbivores are a main determinant of host-plant quality [1], [7], and thus are particularly important to characterize. Heterogeneity in plant defense arises from variation in the particular type of chemical or mechanical defense a plant employs, and from variation in the amount of a given defense expressed in a plant or plant tissue. Chemical and mechanical defenses are hypothesized to differ in their effectiveness against herbivores with different diet breadths. In particular, while many chemical defenses tend to confer resistance against generalist herbivores via their deterrent or toxic effects [8], mechanical defenses, which present a physical barrier, typically confer resistance against both generalists and specialists [9]. The extent to which plants invest in chemical versus mechanical defense depends on many factors including resource availability, herbivore community composition, phylogenetic constraints, and whether defenses have non-redundant functions [10], [11], [12], [13], [14]. While there is evidence that resource-limited plants exhibit trade-offs in investment among chemical and mechanical defenses [15], [16], a recent global survey of more than 260 plant species found that plants often invest highly in both, with little indication of trade-offs [17], [18]. This pattern suggests that natural selection favors the maintenance of an array of defenses with different efficacy against co-existing herbivore guilds [17].

Variation in defense occurs both among and within plants. Among individual plants, variation in maternal lines typically has a genetic basis (though environmentally driven maternal effects can also contribute) [19]. Maternal variation in defenses can be quite pronounced [20], [21], [22], with more highly defended maternal lines experiencing reduced oviposition and feeding damage [21], [22], [23] and higher fitness in the presence of herbivores [13]. Within plants, variation in levels of defenses among different tissues is common [24] [25]. In particular, chemical defenses are often distributed within plants such that young, highly photosynthetic and nutritious (i.e., valuable) leaves are better defended than older (i.e., less valuable) leaves, as predicted by optimal defense theory [24], [25]. This optimized partitioning of defenses in the face of limiting resources appears to be widespread [26], and has been shown to increase plant fitness [27] and structure herbivore foraging patterns [28]. If a particular strategy for deploying defenses among leaves of different age provides a fitness advantage, maternal lines within a population should express convergent strategies.

Here we quantify intra-specific variation in both chemical (iridoid glycosides) and mechanical (trichome length and leaf strength) defenses of common mullein (*Verbascum thapsus* L, Scrophulariaceae; hereafter mullein), an introduced herbaceous species whose ecology and fitness are shaped by co-occurring herbivores [29], [30]. Specifically, damage by generalists (grasshoppers and noctuid cutworms) in the introduced range is common and can be substantial [30], with previous work indicating that protection from herbivores significantly increases mullein's fitness [30]. As such, we examine defenses at two scales of variation that likely shape the outcome of plant-herbivore interactions – among maternal lines within a population and among leaves of different ages within plants – and investigate how this variation affects feeding of a generalist insect herbivore. We asked: 1) Is there significant variation among maternal lines in overall investment in iridoid glycosides (secondary metabolites found in mullein), trichome length, and leaf strength? 2) Do different-aged leaves within a plant vary in levels of investment in these defenses, and is such variation consistent among maternal lines (indicating convergent strategies for how defenses are partitioned among leaves of different value)? 3) At the whole-plant scale, can we detect trade-offs between investment in the different types of defense, or between plant defense and performance? 4) Is the degree of damage caused by a common generalist herbivore, the cabbage looper (*Trichopulsia ni* Hübner, Noctuidae), affected by variation in levels of defense among plants or different-aged leaves within plants? We found significant variation in chemical and mechanical defenses, both among maternal lines and between different-aged leaves. Feeding trials with *T. ni* revealed that this variation is linked to palatability, with caterpillars preferring plants with lower amounts of iridoid glycosides, and older leaves with lower investment in all defenses.

## Materials and Methods

### Study system

Common mullein is a monocarpic perennial (typically biennial) plant that was introduced to North America from Europe in the early 1600s [31], [32]. It is widely distributed in both its native and introduced ranges [31], [33], although it is more abundant across large portions of its introduced range in the western U.S. than it is in Europe [29]. In the introduced range the most important leaf herbivores are generalist grasshoppers and generalist noctuids, which can cause significant damage, especially to older leaves [14], [34]. Introduced mullein is also attacked by an inadvertently introduced specialist thrips (*Haplothrips verbasci* Osborn), which feeds on cell contents, and by an introduced specialist weevil (*Rhinusa tetra* Fabricius), which can destroy large quantities of seed [35].

Mullein employs both chemical and mechanical defenses against leaf herbivory. Two important chemical defense compounds are the iridoid glycosides aucubin and catalpol. These are monoterpene-derived compounds that are present in more than 50 plant families, with demonstrated importance in shaping plant-insect interactions [36], [37]. For example, while iridoid glycosides can act as deterrents to generalist feeders and may interfere with their digestive metabolsim [37], [38], they can also act as attractants to adapted specialists such as buckeye caterpillars (*Junonia coenia* Hübner, Nymphalidae) [39]. In natural mullein populations, young leaves exhibit higher levels of iridoid glycosides, as well as higher proportions of the potentially more toxic compound catalpol, than do old leaves [34]. Moreover, young leaves with high iridoid content are significantly less damaged than older leaves with low iridoid content [34]. Mullein also has conspicuous trichomes that are more prevalent on young than old leaves and reduce palatability to generalist grasshoppers [14]. Leaf strength (often referred to as leaf toughness; see [40] and [41] for a clarification of terms), another structural trait that confers resistance to herbivory [10], [42] and reduces insect performance [43], [44], has not yet been quantified in mullein.

The current study expands upon earlier findings [14], [29], [34] by quantifying levels of both physical and chemical defenses among maternal lines of mullein plants grown under common conditions. This approach tests whether the plant- and leaf-level variation previously observed in the field [34] is constitutive (i.e. is found even in a greenhouse environment without herbivory to induce defenses), and directly links the observed variation in defenses to feeding behavior in a generalist noctuid. *Trichoplusia ni* has a Nearctic distribution that overlaps that of mullein and it occasionally feeds on mullein in nature (Alba, pers. obs.). It also feeds on other iridoid-containing members of the Scrophulariaceae (*Linaria* sp.) and Plantaginaceae (*Plantago* sp.) families [45], recommending it as a suitable choice for exploring feeding responses to these particular chemicals.

### Experimental design

Seeds for the experiment were collected in late summer 2008 from a single mullein population covering ∼300×150 m in Loveland, Colorado (40° 22' 32" N, 105° 13' 34" W; 1658 m.a.s.l.) after obtaining a permit issued by land managers in Larimer County. We focus on characterizing variation in plants from a single population because the individual is the scale at which natural selection on phenotypic traits occurs, and it is the scale at which herbivorous insects choose their hosts. Mullein is a facultative out-crosser, with flowers that, in the absence of cross-pollination, self-fertilize upon closing. As such, seeds collected from any one plant (i.e., maternal line) are either full- or half-sibs. When grown in a common environment, variation in traits attributable to maternal line most likely represents genetic variation, although, given that the seeds were field-collected, variation due to maternal effects is also possible.

Seeds were stored at 4°C until the beginning of the experiment. In February 2010, we sowed several individual seeds from each of 10 maternal lines into a germination tray containing Farfard germination mix and placed the trays on a mist bench (average daytime temp., 24.8°C; average daytime relative humidity, 59.5%; average nighttime temp., 19.9°C; average nighttime relative humidity, 77.4%). Seedlings were thinned as necessary. After 4 weeks, seedlings were transplanted into 1-gallon pots containing Farfard #2 potting soil and moved to a separate greenhouse (average daytime temp., 21.9°C; average daytime relative humidity, 64.5%; average nighttime temp., 18.4°C; average nighttime relative humidity, 72.6%). At the time of transplanting, we measured cotyledon length and width with calipers to obtain an estimate of maternal provisioning for inclusion in subsequent analyses. Plants were fertilized once with 1 tsp. of Osmocote slow-release NPK (14-14-14) fertilizer and watered as needed (every 2–3 days). In May 2010, when plants were 12 weeks old and had ∼12–14 leaves, we harvested five individuals per maternal line for defense measurements and additional individuals of each maternal line for use in feeding trials. In one case (maternal line 5), only three individuals were available for defense measurements.

### Defense measurements

At harvest, we separated rosettes into different-aged leaves. Mullein rosettes have leaves in ranks of two, with each rank growing perpendicular to the successively younger and older ranks. Nearly all rosettes were composed of 6 or 7 ranks of leaves, of which we designated the innermost 3 ranks as “young”, the 4^th^ and 5^th^ ranks as “medium”, and the 6^th^ and 7^th^ ranks as “old”. These designations were based on clear qualitative differences between leaves of different maturity, including the degree of pubescence and leaf color. The oldest leaves were not yet senescent because the plants were young.

#### Iridoid glycosides

Leaves were separated by age class for analysis of percent aucubin, percent catalpol, percent total iridoid glycosides, and the proportion of iridoids composed of catalpol. The same leaves used to measure trichome length and leaf strength (detailed below) were used for chemical analysis. Leaves were oven-dried at 50°C to a constant mass and weighed to the nearest 0.01 g. Samples were ground into a fine powder from which we removed trichomes by passing samples over a mesh screen. We prepared 50-mg subsamples for chemical extraction and analysis by gas chromatography [20]. Subsamples were extracted in methanol and the remaining tissue filtered off under a vacuum. We added an internal standard (phenyl-β-D-glucopyranoside  =  PBG) to the extract, evaporated the methanol, and partitioned the residue between water and ether to remove chlorophyll and hydrophobic compounds (found in ether layer). An aliquot of the remaining aqueous solution was evaporated and derivatized with Tri-Sil-Z (Pierce Chemical Company) prior to injection into a Agilent 7890A gas chromatograph (Agilent Technology) using an Agilent DB-1 column (30 m, 0.320 mm, 0.25 µm particle size). Concentrations of these compounds were quantified using ChemStation B-03-01 software and they are presented as percent dry weight for comparative purposes [46].

#### Trichome length and leaf strength

We measured trichome length and leaf strength on freshly harvested leaves from each age group. Young leaves were always of rank 3, medium leaves were predominantly of rank 5 (and occasionally 4), and old leaves were predominantly of rank 6 (and occasionally 7). Half of each leaf was randomly assigned to trichome measurements and the other half assigned to leaf strength measurements. We made 3 measurements of trichome length and leaf strength per leaf and averaged these numbers together for analysis. For all measurements we took care to avoid major veins, and located the sampling positions consistently both within and among leaves. Trichome length was measured using an ocular micrometer at 40× magnification [14]. Using a hole-punch, we removed a 0.6-cm-diameter circle of tissue, held the circle gently on edge with tweezers, and measured the length of trichomes from the epidermal layer out. The length of the trichomes was taken to be the dominant layer or mat of hairs and did not include the occasional longer hair [14].

Leaf strength measurements were made using a Lloyd LF-Plus universal testing machine customized to work as a leaf penetrometer. The penetrometer pushes a circular probe (7.1 mm^2^) through the leaf at a constant speed, measuring the force applied to the probe continuously with a 20 Newton load cell, accurate to within 1% of the force measurement. We report the maximum amount of force per unit area applied when puncturing the leaf in kN/mm^2^. This mechanical property is the equivalent of leaf “strength”, as opposed to “toughness”, [39], [41], [42], [47], with these two terms often treated (erroneously) inter-changeably in the literature. In precise terms, strong leaves limit an herbivore's ability to achieve the stress required to initiate a crack, while tough leaves require more work to extend a crack once initiated [41], [42].

Feeding trials: Effect of leaf age, overall levels of defense, and trichomes on palatability

To determine how leaf age and variation in overall levels of defense (i.e., when averaging over leaf age) affected feeding preferences of a generalist caterpillar species, we used *Trichoplusia ni* (cabbage looper) and set up paired choice tests. Young and old leaves from the maternal lines for which we measured defenses were placed in a square petri dish (23 cm×23 cm) and offered to 4^th^-instar caterpillars raised on artificial medium (obtained from Bio-Serv Entomology Division, Frenchtown, New Jersey). To help leaves maintain their turgor pressure, we used moist paper towels to line each dish and wrap leaf petioles. Each dish was then sealed with Parafilm. To avoid biasing caterpillar choice based on leaf size, the total area of young and old leaves offered to caterpillars was set up to be similar by pairing 4 young leaves with two old leaves. The feeding experiments were conducted in two blocks (n = 10 plants on each of two separate dates) under similar conditions. In the first block, individuals of only 7 of the 10 maternal lines were available for inclusion, thus 3 maternal lines were represented twice. In the second block, all 10 maternal lines were represented. The first trial lasted 24 hours and the second trial lasted 48 hours because caterpillars in the second block fed at a slower rate. At the end of each trial, all leaves were scanned and the missing leaf area was quantified with WinFolia software (Regent Instruments Inc.). Because larvae were raised on an artificial medium, there was no induction of feeding preferences for mullein. The results thus best estimate the behavior of naïve foragers that lack any induced detoxifying enzymes that could modulate feeding behavior [48]. Given the highly mobile nature of *T. ni* and its wide diet breadth, such naïve behavior might be expected under field conditions.

We also tested whether trichomes affected the palatability of leaves to cabbage loopers using choice tests in which one half of a young mullein leaf (rank 3) was shaved and the other half left intact. (We used only young leaves in this experiment because they are heavily covered in trichomes and are the most relevant age class for detecting trichome effects on palatability.) We again used 4^th^-instar caterpillars (n = 10) and the same maternal lines used for measuring defenses, and prepared each dish with moist paper towels and Parafilm as detailed above. The trial lasted for 24 hours, after which time all leaves were scanned and damage quantified using WinFolia.

### Statistical Analysis

All statistical analyses were conducted in SAS (v. 9.2, SAS Institute 2004). For iridoid glycoside analysis, we first conducted a Pearson's correlation test to determine whether investment in aucubin and catalpol was related. This test indicated no correlation [correlation coefficient  = 0.12, *P* = 0.16], precluding the need to conduct MANOVA. As a result, we used univariate ANOVA to analyze the effects of leaf age, maternal line, and the interaction of leaf age and maternal line on iridoid glycoside content (aucubin, catalpol, total iridoid glycosides, and the proportion of iridoids composed of catalpol), as well as trichome length and leaf strength, respectively. Leaf age was treated as a fixed effect and maternal line and its interaction with leaf age were treated as random effects. We also included a “repeated” statement (replicate × leaf age nested within maternal line) to indicate that young and old leaves from the same plant were repeated measures on that plant. The significance of the random effects was determined by calculating the difference between the residual log-likelihood of the full model (with random effects included) and the reduced model(s) (with random effects sequentially removed). The significance of these test statistics was evaluated against a chi-square distribution with 1 degree of freedom (Table 1) [49]. Cotyledon size and rosette weight were also included as covariates to explore whether they explained additional variation in defense investment. Cotyledon size did not explain additional variation for any response variable and was removed from the models. Rosette weight explained additional variation in trichome length (*F*_1,113_ = 6.1, *P* = 0.01), and was retained in that model. Iridoid glycoside levels were arcsine-square root transformed and trichome length and leaf strength were square-root transformed to improve normality and homogeneity of variance.

**Table 1:** ANOVA results of the effect of leaf age, maternal line (ML), and the interaction of leaf age and maternal line on the percent dry weight of the iridoid glycosides aucubin and catalpol, the percent dry weight of total iridoid glycosides (aucubin + catalpol), the proportion of iridoids composed of catalpol, trichome length, and leaf strength.

	Aucubin		Catalpol		Total IG		Proportion Catalpol		Trichome Length		Leaf Strength	
**Fixed Effect**	*F(df)*	*P*	*F(df)*	*P*	*F(df)*	*P*	*F(df)*	*P*	*F(df)*	*P*	*F(df)*	*P*
Leaf Age	0.65(2,18)	0.53	**7.4(2,18)**	**0.005**	3.3(2,18)	0.06	**12(2,18)**	**0.0005**	**177(2,18)**	**<0.0001**	**3.9(2,18)**	**0.04**
**Random Effects**	?^2^	*P*	?^2^	*P*	?^2^	*P*	?^2^	*P*	?^2^	*P*	?^2^	*P*
ML	**5.3**	**0.01**	**4.6**	**0.02**	**6.9**	**0.004**	0	0.5	**6.9**	**0.004**	**13.5**	**0.0001**
ML x leaf age	**3.3**	**0.03**	0	0.5	0	0.5	0.0	0.5	0	0.5	0	0.5

Significant terms are in bold.

To explore whether chemical and mechanical defenses and plant performance (biomass) were positively or negatively correlated, we generated Pearson's correlation coefficients using the “proc corr” procedure in SAS. Defense levels were averaged over leaf age.

We used ANOVA with leaf age and block (date of feeding trial) as main effects to determine whether *T. ni* larvae preferred young or old leaves. As with the models constructed for the defense measurements, we included a repeated measures statement (leaf age × maternal line within block) to account for the non-independence of young and old leaves from a given plant. We explored both the absolute area of each leaf consumed (initial area – final area; square-root transformed) and the proportion of leaf area consumed (initial area – final area/initial area; arcsine-square-root transformed). Both response variables produced similar patterns (see Results), and we present only the data for absolute area. To test for the effect of the presence or absence of trichomes on *T. ni* feeding preferences, we used a paired t-test on untransformed data.

To explore whether *T. ni* feeding preferences are linked with variable levels of defense investment among plants, we used multiple regression analysis. The response variable was the square-root transformed absolute leaf area eaten for a particular maternal line, averaging over the area removed from both young and old leaves. We estimated the total leaf area removed per plant because, in terms of herbivore effects on fitness, the individual plant is the proper scale for analysis. The predictor variables were the average investment in defense of the five replicates per maternal line (3 replicates in the case of ML 5). We included all six defensive measures (percent aucubin, percent catalpol, percent total iridoid glycosides, proportion of the total iridoid glycosides composed of catalpol, trichome length, and leaf strength) in the model and conducted concomitant forward and backward stepwise regression. The significance level for each variable's entry and retention in the model was 0.15, which is the default level in SAS. For this analysis, we used only the data from the second feeding trial in which all 10 maternal lines were represented. We removed a single outlier associated with maternal line 8 based on graphical analysis of the model residuals and qq-plot (reducing the number of replicates for maternal line 8 to four in this analysis). Because we were not able to quantify defenses of the plants used in the feeding trials, we assume that their defense levels were comparable to those estimated for the other individuals of the same maternal line.

## Results

### Iridoid glycosides

**Figure 1:**
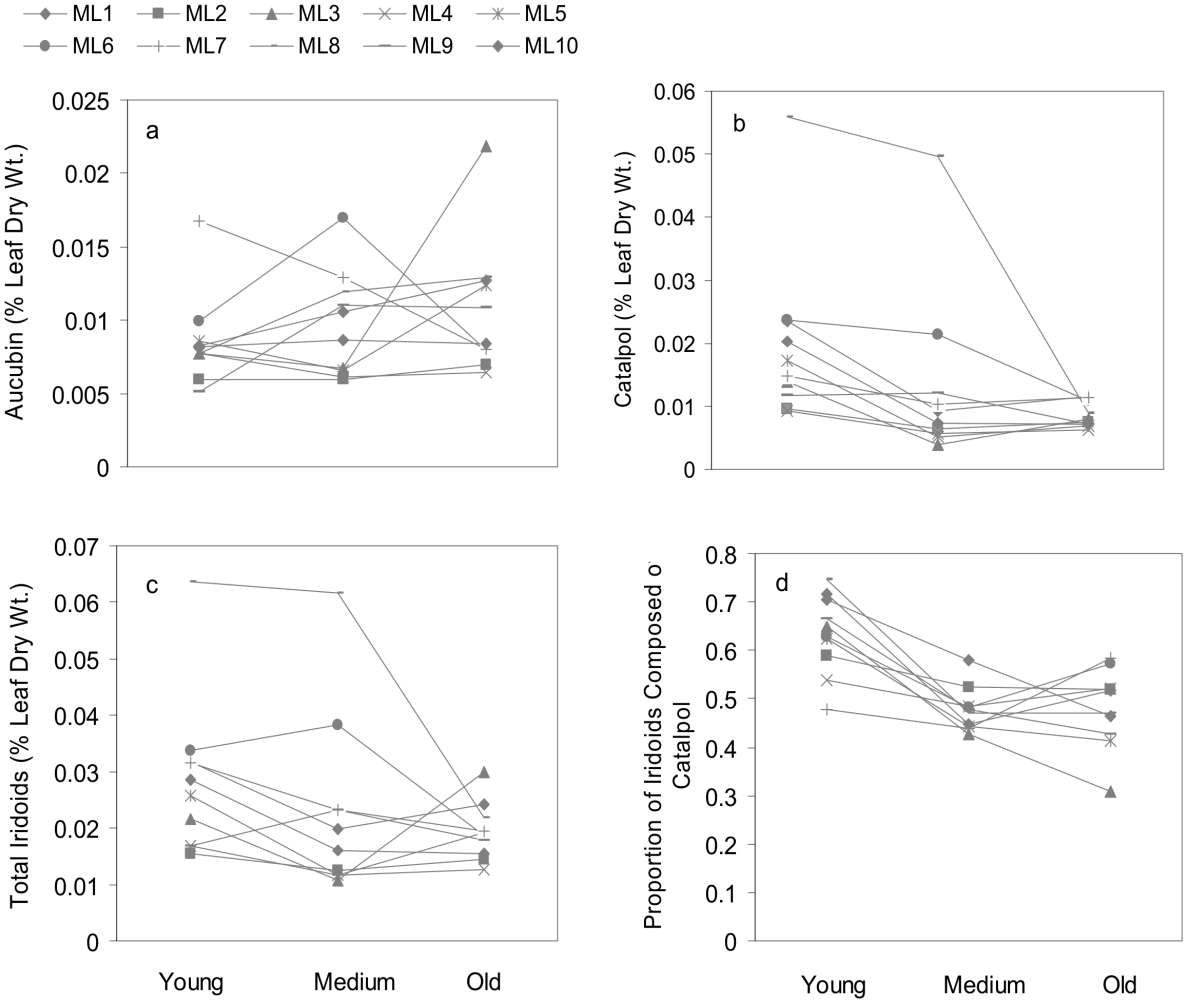
Iridoid glycoside levels in *Verbascum thapsus*. Untransformed means of the percent leaf dry weight of a) aucubin, b) catalpol, c) total iridoids glycosides, and d) the proportions of iridoids composed of catalpol in young, medium, and old leaves of 10 maternal lines (M1-M10) of *Verbascum thapsus* (common mullein). Reaction norms show lines connecting maternal lineages. Standard error bars are omitted for clarity and are presented in Tables S5-S8.

Overall, the detected levels of iridoid glycosides were low (Figure 1, a-c), possibly because rosettes were harvested at only 12 weeks of age, or due to other factors that can lead to low levels of carbon-based secondary compounds, such as low light or high nutrient availability [18]. The percent of leaf dry weight composed of aucubin was consistently lower than the percent composed of catalpol (cf. Figures 1a and 1b). Maternal lines were an important source of variation in iridoid investment, significantly affecting the amounts of aucubin, catalpol and total iridoid glycosides present when averaging across leaf age (Figure 1, a-c; Table 1). In contrast, the proportion of iridoids composed of catalpol was consistent among maternal lines (Figure 1d; Table 1).

The percent dry weight of aucubin did not consistently differ with respect to leaf age (Figure 1a; Table 1). All other measures of iridoid investment declined with leaf age, with the percent leaf dry weight of catalpol and the proportion of iridoids composed of catalpol exhibiting the strongest differentiation (Figure 1b and 1d; Table 1). The percent dry weight of total iridoid glycosides (aucubin + catalpol) showed a marginally significant decline with age (Figure 1c; Table 1). Differences of least square means (results not shown) revealed that significant differences in investment in catalpol, total iridoids, and the proportion of iridoids composed of catalpol were restricted to comparisons of young versus medium-aged, and young versus old leaves, while medium-aged and old leaves did not differ in any case.

Finally, there was a significant leaf age by maternal line interaction associated with the percent dry weight of aucubin (Figure 1a; Table 1), indicating that the amount of aucubin present in different-aged leaves varied by maternal line. For example, some maternal lines showed a clear decrease in aucubin with leaf age (maternal line 7, Figure 1) while others showed an increase (maternal line 8, Figure 1) or no clear pattern (maternal line 2, Figure 1).

### Trichomes and leaf strength

**Figure 2:**
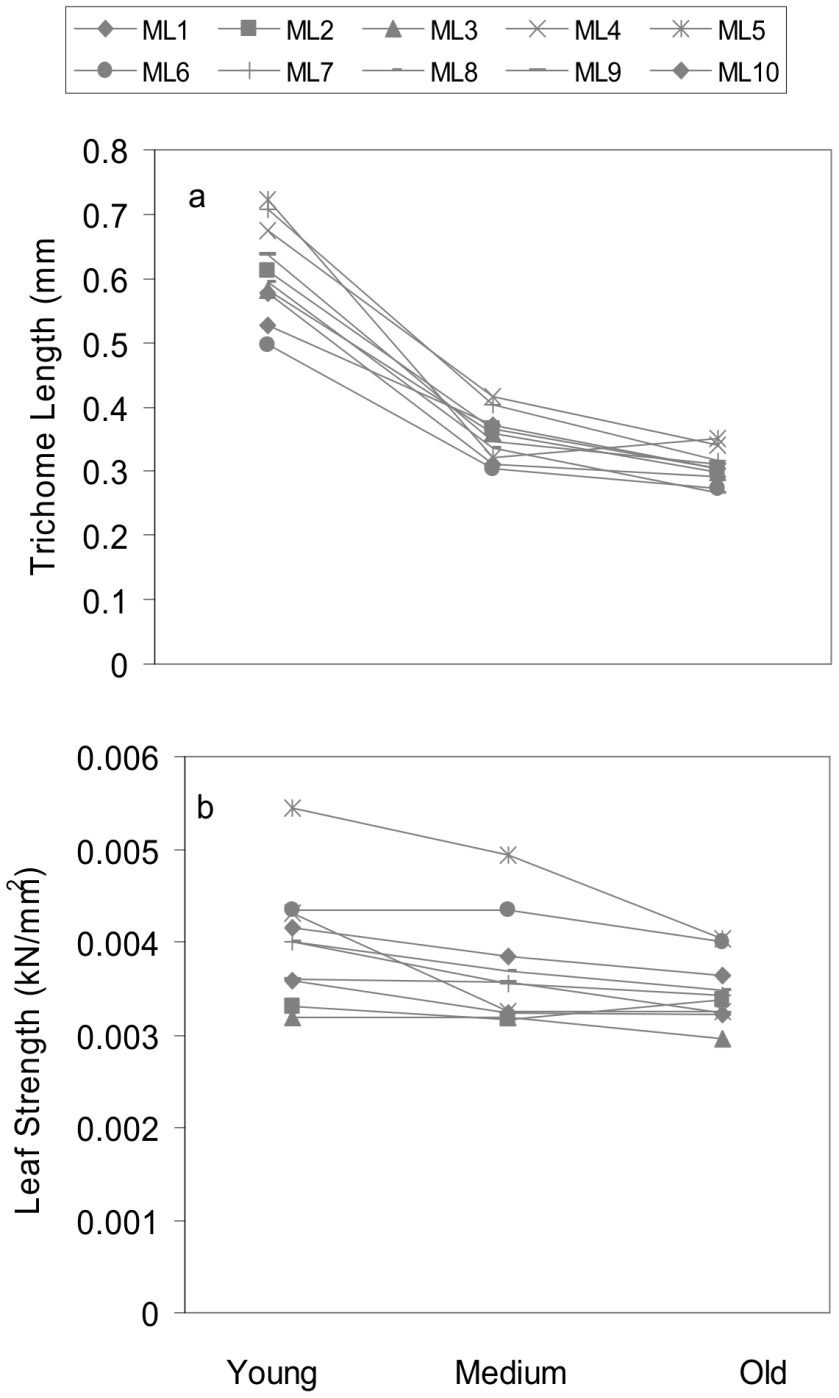
Structural defense levels in *Verbascum thapsus*. Untransformed means of a) trichome length and b) leaf strength (expressed as the force/area [kN/mm^2^] required to puncture a leaf using a penetrometer) in young, medium, and old leaves of 10 maternal lines (M1-M10) of *V. thapsus*. Reaction norms show lines connecting maternal lineages. Standard error bars are omitted for clarity and are presented in Tables S9 and S10.

Both trichomes and leaf strength showed pronounced variation with respect to maternal lines and leaf age (Figure 2a, b; Table 1). For trichomes, differences of least square means indicated that all leaf ages were significantly differentiated from one another, with young, medium, and old leaves exhibiting successively shorter trichomes (young vs. old leaves, t_113_ = 17.1, *P*<0.0001; young vs. medium leaves, t_113_ = 14, *P*<0.0001; medium versus old leaves, t_113_ = 3.1, *P* = 0.006). For leaf strength, only young and old leaves were significantly different, with young leaves being significantly stronger (t_113_ = 2.7; *P* = 0.02). Neither structural defense showed a significant leaf age by maternal line interaction (Figure 2a, b; Table 1), indicating consistent deployment among families with respect to leaf age. See Table S1 for defense data.

### Correlations between chemical and mechanical defense, and defense and performance

When averaging over leaf age, there were no significant relationships between any aspect of the iridoid glycoside profile and mechanical defense, between the two mechanical defenses, or between any defense and plant biomass (Table S2).

### Feeding trials: Effect of leaf age, overall levels of defense, and trichomes on palatability

**Figure 3:**
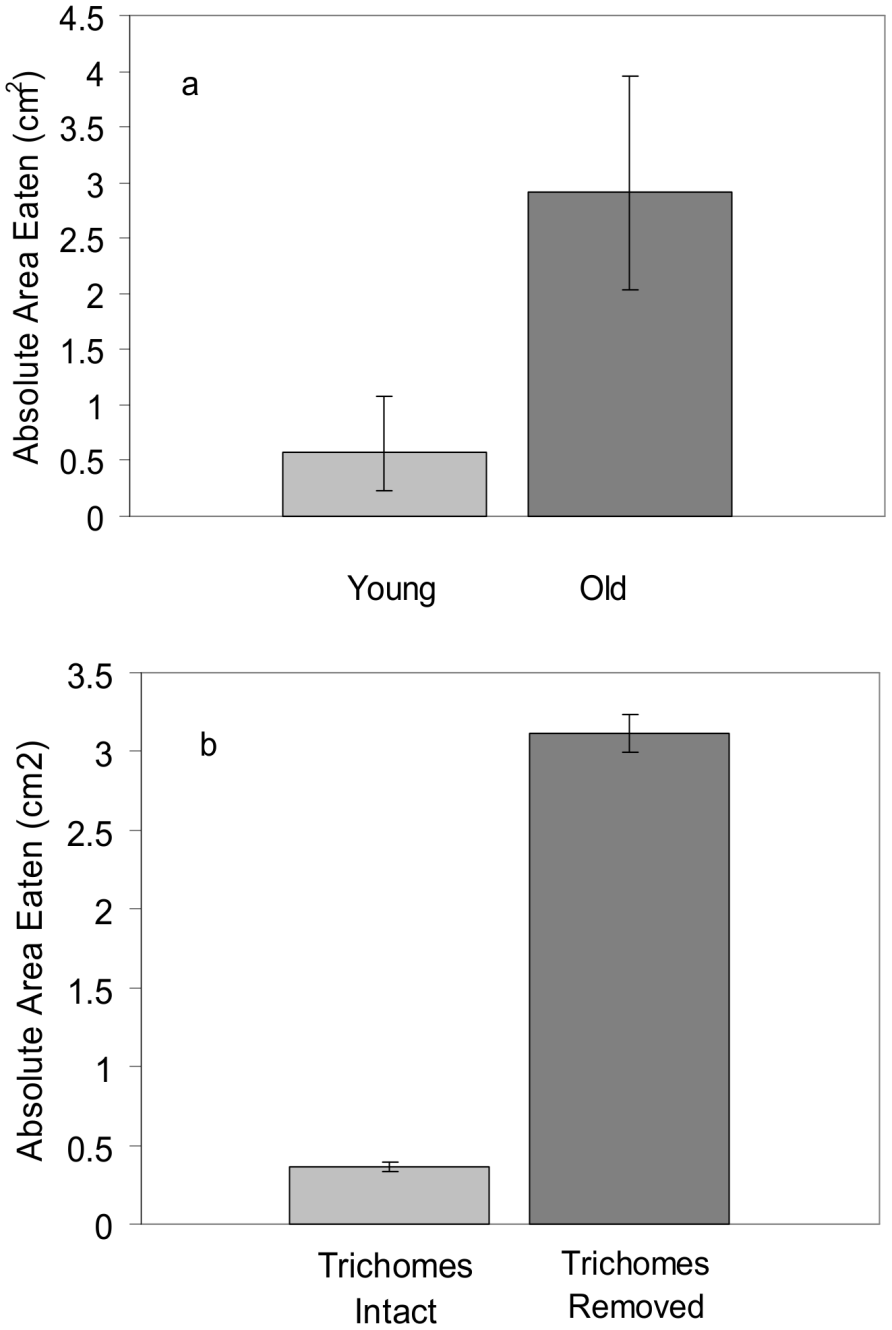
Consumption of *Verbascum thapsus* leaves by a generalist herbivore. Means and standard errors of the absolute leaf area consumed (in cm^2^) by *Trichoplusia ni* (cabbage looper) larvae when presented with a choice between a) young and old leaves (*P* = 0.001) of *V. thapsus* and b) a single young leaf, half of which had intact trichomes, and half of which had trichomes removed with a razor blade (*P*<0.0001). Means for the young/old comparison are back-transformed. Means for trichomes intact/removed are un-transformed. See Tables S3 and S4 for feeding trial data.

**Figure 4:**
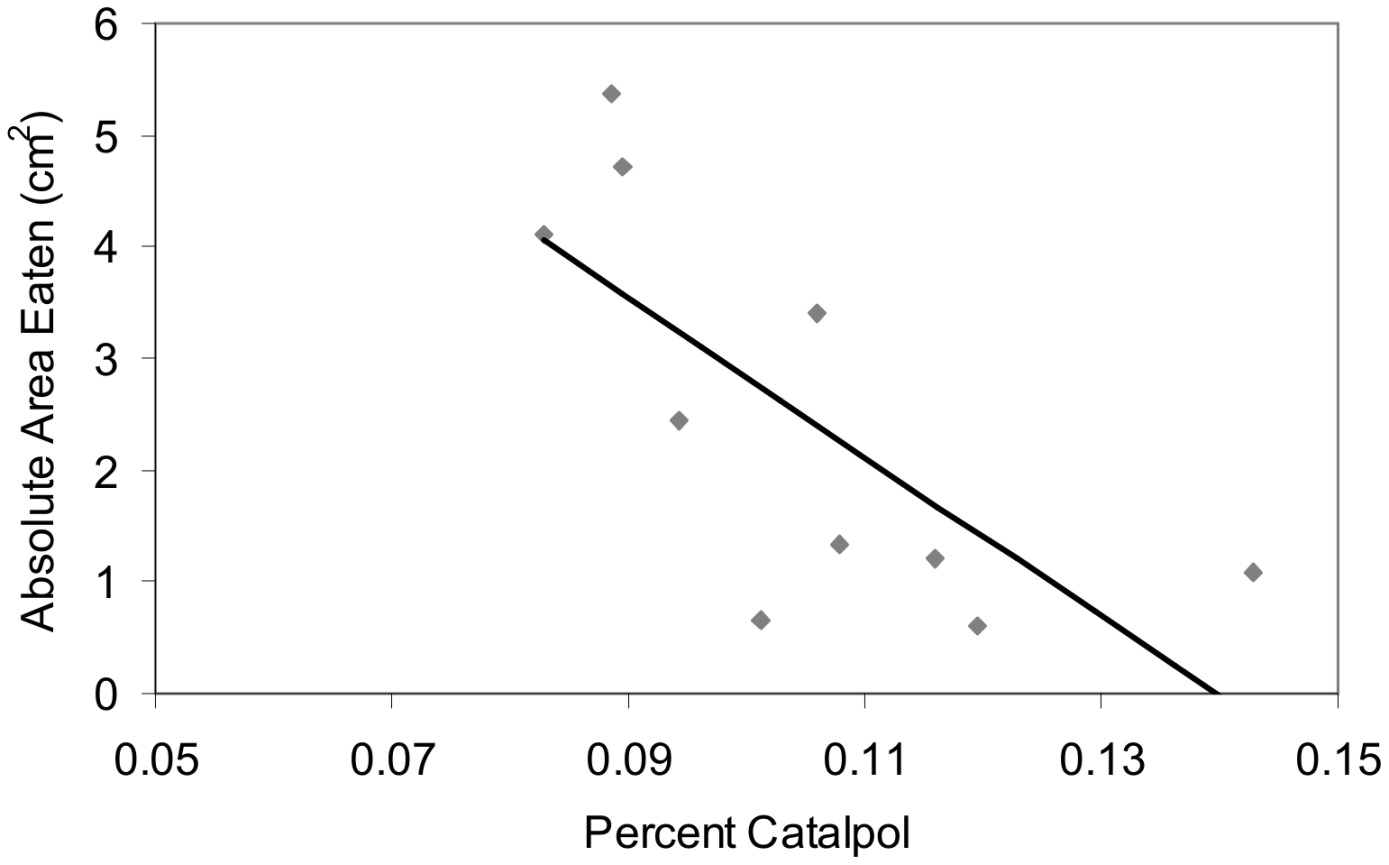
Relationship between feeding damage and chemical defense in *Verbascum thapsus*. Scatterplot showing the relationship between *Trichoplusia ni* feeding damage to *V. thapsus* and different levels of percent leaf dry weight (arcsin-square-root transformed) composed of catalpol. This defense explained the most variation in caterpillar damage to plants when averaging levels of defense over leaf age (see text for details of the statistical approach).

We found that *Trichoplusia ni* significantly preferred old leaves to young (Figure 3a; *F*_1,37_ = 11.73, *P* = 0.0015) and leaf tissue with trichomes removed to leaf tissue with trichomes intact (Figure 3b; t_10_ = −7.04; *P*<0.0001). The same patterns were found when the proportion leaf area consumed was used as the response variable instead of total area consumed (old versus young leaves, *F*_1,37_ = 6.3, *P* = 0.017; leaf tissue with trichomes removed versus trichomes intact, t_10_ = 7.8; *P*<0.0001). Overall variation in defense (when averaging over leaf age) among individual plants explained significant variation in feeding damage due to the negative effect of the percent leaf dry weight of catalpol (*R* ^2^ = 0.51, *F*_1,8_ = 8.25, *P* = 0.02; Figure 4b), which was the only defense characteristic retained in the multiple regression model following stepwise deletion tests. See Tables S3 (young versus old leaves) and S4 (trichome removal) for feeding trial data.

## Discussion

Working within a single population of a common introduced forb, we found that not only do levels of herbivore defense vary, but that this variation in defense matters, with more heavily defended plants and leaves avoided by a generalist insect herbivore. Phenotypic variation in defense investment among plants can influence herbivore distribution and abundance, and if genetically based, represents the raw material on which selection can act, driving adaptive shifts. Here, we demonstrate that both chemical and mechanical defense vary among maternal lines grown in a common environment. Although we cannot rule out a role for environmentally driven maternal effects in shaping the observed phenotypes [19], the fact that maternal provisioning (cotyledon size) does not explain variation in the traits of interest, and that variation in iridoid glycosides and trichomes is heritable in other systems [50], [51], [52], [53], support the hypothesis that variation among maternal lines is genetically based. Thus, with the exception of the proportion of total iridoids composed of catalpol, this population exhibits the potential for adaptive evolution in defenses. Further, the results highlight that herbivores on mullein encounter both quantitative and qualitative sources of host-plant heterogeneity, in that two disparate classes of defense, chemical and structural, vary in degree among maternal lines. If such intra-specific variation in defense leads to host-switching behavior [3], [54], it could in turn affect the amount and distribution of herbivory among plants within the population [3] and, ultimately, plant fitness.

The differentiation among maternal lines in percent aucubin and catalpol (with coefficients of variation [CV] of 22% and 45%, respectively) stands in contrast to the proportion of total iridoid glycosides composed of catalpol, which was consistent (CV of 9%) and high across maternal lines. This pattern, which reflects low levels of aucubin relative to catalpol, resulting in catalpol driving the overall levels of total iridoid glycosides, could arise in at least two ways. First, given that catalpol is potentially more toxic than aucubin [36], [38], generalist herbivores may react more strongly to its percent dry weight than that of aucubin or the total percent iridoid glycosides, potentially selecting for increasing mean levels of catalpol in the population while at the same time reducing variation in the proportion of iridoids composed of catalpol. Indeed, our feeding trials suggest that for at least one generalist herbivore, percent dry weight of catalpol most strongly determines palatability among different plants. Second, low levels of aucubin could arise from its rapid conversion into catalpol, for which it serves as the biosynthetic precursor [55]. In this case, a lack of differences among maternal lines in the proportion of catalpol may simply reflect the mechanics of the biosynthetic pathway, which could arise independently of natural selection by herbivores.

There is a marked consistency with which maternal lines better defend young than old leaves, with percent aucubin being the only defense with significant genetic variation among maternal lines in its allocation by leaf age. These findings support the predictions of optimal defense theory, and corroborate other studies where chemical defenses are more abundant in young (i.e., highly nutritious or photosynthetic, and thus more valuable) versus old leaves [26], including studies quantifying aucubin and catalpol in *Plantago* spp. [20], [56]. This finding is in accordance with previous work on mullein, showing higher levels of iridoids in young versus old leaves of field-grown mullein in the introduced range [34] and confirms that the age-related defense distributions are constitutive, because plants in the current study were grown in the absence of herbivory. The overall levels of total iridoids in the field-grown plants are higher (averaging up to ∼10% dry weight in young leaves and >2% weight in old leaves) than the levels observed here [34], possibly because the plants in the present experiment were young and small at harvest, or iridoid production was reduced under greenhouse conditions (e.g., due to lighting or nutrient availability). Nonetheless, the qualitative pattern of young leaves being more highly defended than old leaves is expressed in both the natural and controlled settings.

We additionally found that young leaves are better defended by the two structural defenses. This class of defenses has been less explored in the framework of optimal defense theory (but see [14]). This is possibly because while the main role of plant secondary metabolites is highly likely to be defense, and thus herbivores the main agent of selection, mechanical structures can serve other roles [14] or may vary simply as a by-product of the mechanics of leaf growth (e.g., trichomes can become less dense as leaf surface area expands) [57]. Regardless of the agent of selection on mechanical defenses, there is good evidence that they deter herbivory. For example, herbivore feeding damage declines with increasing trichome density among natural plant populations [58], crop cultivars [59], and individual plants [51], [60]. While fewer studies have quantified differences in trichome investment related to leaf age, those that have similarly report a decrease in trichome density as leaves age [61], [62], with herbivores responding positively or negatively to such differences depending on their identity.

In terms of leaf strength, our results contrast with other studies that have reported higher strength in old relative to young leaves, in many cases associated with herbivores avoiding or performing poorly on older leaves [10], [63], [64], [65], [66], [67]. An increase in strength as leaves age is in part due to the differentiation of cells into support tissues such as sclerenchyma [42]. External structures such as thorns, spines, and hairs (i.e., trichomes) can also effectively increase leaf strength as measured here [41]. The pattern of declining strength with leaf age could thus be due to young leaves being significantly more pubescent than old leaves, a pattern found in other plant species with highly pubescent young leaves (e.g., *Dendrocnide excelsa*) [63]. However, the correlation analysis suggests that leaf strength is not entirely redundant with pubescence (Table S1). As such, other aspects of leaf anatomy such as leaf thickness or tissue density appear to contribute to the higher leaf strength in young leaves, with strong leaves contributing another potential deterrent to foraging herbivores.

We found no evidence for a trade-off between investment in chemical versus mechanical defense, between the two types of mechanical defense, or between levels of defense and plant performance (biomass). Because we used family means to assess the relationships, we can infer a lack of genetic correlation among investment in the different defenses. Although the number of maternal lines used here was relatively low, affording us less power for these tests, the lack of measurable trade-offs between defense traits, in conjunction with the variation among traits, suggests that they could independently respond to selection. It also suggests that the different types of defense serve unique roles in protecting plants [17], since it can be hypothesized that redundant defenses would be inefficient and therefore selected against. The lack of trade-offs found herein mirrors previous findings based on phenotypic correlations (i.e., those generated without family means) across individuals from 14 populations of mullein [68]. In that study, there was no relationship between any aspect of the iridoid profile and trichome length, and in fact there was a positive relationship between percent total iridoids and leaf strength. Additionally, iridoid glycosides and leaf strength were both positively correlated with plant biomass, i.e., larger plants were better defended, providing no evidence for a “grow-or-defend” constraint [69]. A similar lack of trade-offs between iridoid levels and plant growth was also found in *Plantago lanceolata* [56]. Although these findings suggest a lack of constraints on mullein's ability to highly invest in more than one type of defense, the plants studied here were grown in a low-stress environment (e.g., lack of both competition and herbivory), and trade-offs might be more apparent under more realistic conditions [70].

The preference of *T. ni* for older rather than younger leaves shows that the caterpillars' feeding behaviors map onto within-plant variation in defense in a predictable way. Because levels of defense co-vary among leaf ages (i.e., all of the defenses decline with age), it is difficult to parse the relative contribution of each in shaping *T. ni*'s preference for older leaves. This preference reflects patterns observed in the field, where damage from a wider variety of generalist herbivores present in the introduced range was estimated [34]. The negative relationship between feeding damage and percent catalpol corresponds with a previous study showing that catalpol is more toxic to the noctuid *Spodoptera eridania* than aucubin (assuming a correlation between toxicity and palatability [36], [71]). It is surprising that variation in trichome length did not exhibit a relationship with feeding damage, despite the clear preference of *T. ni* for leaf tissue without trichomes. It is possible that the range of variation among different plants is too subtle (relative to leaf age or the even more marked variation of trichomes simply being present or absent) to elicit a behavioral response. Further controlled trials with a wider range of herbivores with various feeding modes and degrees of specialization are needed to fully assess which defenses deter herbivores and under what conditions.

The seeds used in this study were collected from a mullein population in which herbivores are known to dramatically lower plant fitness, reducing seed output an average of 50 percent [30]. Here we established that plants from this site exhibit variation in defense that is maintained when grown from seed in a common environment, and that patterns of defense can be directly linked to the feeding preferences of a widespread generalist herbivore. The fact that young leaves are consistently well defended suggests that herbivory, particularly to young leaves, has influenced fitness, and therefore selection, in the past. Variation among maternal lines demonstrates the potential for selection to act on defenses in the future. Such variation may help this short-lived species to adjust its defenses to the range of herbivore interactions it experiences over its broad geographic range. This suggests an important role for defense phenotypes in shaping both plant and herbivore population dynamics at this site, and supports the broader hypothesis that host-plant quality is central to shaping ecological communities.

## Supporting Information

Table S1 **Raw data used to generate defense and plant performance means for the 10 maternal lines of** ***Verbascum thapsus*** **.**
(XLS)

Table S2 **Pearson's Correlation Coefficients and associated** ***P*** **-values (in parentheses following coefficient).**
(XLS)

Table S3 **Raw data for feeding trials to determine the preference of** ***Trichoplusia ni*** **for young and old leaves of** ***Verbascum thapsus*** **.**
(DOC)

Table S4 **Raw data for feeding trials to determine the preference of** ***Trichoplusia ni*** **for** ***Verbascum thapsus*** **leaves with and without trichomes.**
(DOC)

Table S5 **Untransformed means and standard errors of aucubin expressed in young, medium, and old leaves of** ***Verbascum thapsus*** **.**
(XLS)

Table S6 **Untransformed means and standard errors of catalpol expressed in young, medium, and old leaves of** ***Verbascum thapsus*** **.**
(XLS)

Table S7 **Untransformed means and standard errors of the percent total iridoid glycosides expressed in young, medium, and old leaves of** ***Verbascum thapsus*** **.**
(XLS)

Table S8 **Untransformed means and standard errors of the proportion of iridoid glycosides composed of catalpol expressed in young, medium, and old leaves of** ***Verbascum thapsus*** **.**
(XLS)

Table S9 **Untransformed means and standard errors of trichome length expressed in young, medium, and old leaves of** ***Verbascum thapsus*** **.**
(XLS)

Table S10 **Untransformed means and standard errors of leaf strength expressed in young, medium, and old leaves of** ***Verbascum thapsus*** **.**
(XLS)

## References

[1] Awmack CS, Leather SR (2002) Host plant quality and fecundity in herbivorous insects. Annu Rev Entomol 47: 817–844. 11729092 10.1146/annurev.ento.47.091201.145300

[2] Maddox GD, Root RB (1990) Structure of the encounter between goldenrod (*Solidago altissima*) and its diverse insect fauna. Ecology 71: 2115–2124.

[3] Whitham TG, Young WP, Martinsen GD, Gehring CA, Schweitzer JA, et al (2003) Community and ecosystem genetics: A consequence of the extended phenotype. Ecology 84: 559–573.

[4] Mody K, Unsicker SB, Linsenmair KE (2007) Fitness related diet-mixing by intraspecific host-plant-switching of specialist insect herbivores. Ecology 88: 1012–1020. 17536716 10.1890/06-1338

[5] Hare JD (1992) Effects of plant variation on herbivore-natural enemy interactions. In: Fritz RS, Simms EL, editors. Plant Resistance to Herbivores and Pathogens: Ecology, Evolution, and Genetics.University of Chicago Press, Chicago and London.

[6] Bottrell DG, Barbosa P, Gould F (1998) Manipulating natural enemies by plant variety selection and modification: A realistic strategy? Annu Rev Entomol 43: 347–367. 15012394 10.1146/annurev.ento.43.1.347

[7] Kessler A (2004) Silencing the jasmonate cascade: Induced plant defenses and insect populations. Science 306: 2042–2042. 15232071 10.1126/science.1096931

[8] vander Meijden E (1996) Plant defence, an evolutionary dilemma: Contrasting effects of (specialist and generalist) herbivores and natural enemies. Entomol Exp Appl 80: 307–310.

[9] Müller-Schärer H, Schaffner U, Steinger T (2004) Evolution in invasive plants: implications for biological control. Trends Ecol Evol 19: 417–422. 16701299 10.1016/j.tree.2004.05.010

[10] Coley PD (1983) Herbivory and defensive characteristics of tree species in a lowland tropical forest. Ecol Monogr 53: 209–233.

[11] Steward JL, Keeler KH (1988) Are there trade-offs among antiherbivore defenses in *Ipomoea* (Convolvulaceae)? Oikos 53: 79–86.

[12] Menken SBJ (1996) Pattern and process in the evolution of insect-plant associations: *Yponomeuta* as an example. Entomol Exp Appl 80: 297–305.

[13] Mauricio R, Rausher MD (1997) Experimental manipulation of putative selective agents provides evidence for the role of natural enemies in the evolution of plant defense. Evolution 51: 1435–1444. 28568624 10.1111/j.1558-5646.1997.tb01467.x

[14] Woodman RL, Fernandes GW (1991) Differential mechanical defense: Herbivory, evapotranspiration, and leaf hairs. Oikos 60: 11–19.

[15] Twigg LE, Socha LV (1996) Physical versus chemical defence mechanisms in toxic *Gastrolobium* . Oecologia 108: 21–28. 28307729 10.1007/BF00333210

[16] Hanley M, Lamont B (2002) Relationships between physical and chemical attributes of congeneric seedlings: How important is seedling defence? Funct Ecol 16: 216–222.

[17] Moles AT, Peco B, Wallis IR, Foley WJ, Poore AG, et al (2013) Correlations between physical and chemical defenses in plants: tradeoffs, syndromes, or just many different ways to skin a herbivorous cat? New Phytol 198: 252–263. 23316750 10.1111/nph.12116

[18] Koricheva J, Larsson S, Haukioja E, Keinanen M (1998) Regulation of woody plant secondary metabolism by resource availability: hypothesis testing by means of meta-analysis. Oikos 83: 212–226.

[19] Rasman S, de Vos M, Jander G (2012) Ecological role of transgenerational resistance against biotic threats. Plant Signal Behav 4: 447–449. 10.4161/psb.19525 PMC3419029 22499174

[20] Bowers MD, Stamp NE (1993) Effects of plant age, genotype, and herbivory on *Plantago* performance and chemistry. Ecology 74: 1778–1791.

[21] Mauricio R (1998) Costs of resistance to natural enemies in field populations of the annual plant *Arabidopsis thaliana* . Am Nat 151: 20–28. 18811421 10.1086/286099

[22] Handley R, Ekbom B, Agren J (2005) Variation in trichome density and resistance against a specialist insect herbivore in natural populations of *Arabidopsis thaliana* . Ecol Entomol 30: 284–292.

[23] Biere A, Marak HB, van Damme JMM (2004) Plant chemical defense against herbivores and pathogens: Generalized defense or trade-offs? Oecologia 140: 430–441. 15146326 10.1007/s00442-004-1603-6

[24] Mckey D (1974) Adaptive patterns in alkaloid physiology. Am Nat 108: 305–320.

[25] Rhoades DF (1976) Toward a general theory of plant antiherbivore chemistry. Recent Adv Phytochem 10: 168–213.

[26] McCall AC, Fordyce JA (2010) Can optimal defence theory be used to predict the distribution of plant chemical defences? J Ecol 98: 985–992.

[27] Ohnmeiss TE, Baldwin IT (2000) Optimal Defense theory predicts the ontogeny of an induced nicotine defense. Ecology 81: 1765–1783.

[28] Zangerl AR, Rutledge CE (1996) The probability of attack and patterns of constitutive and induced defense: A test of optimal defense theory. Am Nat 147: 599–608.

[29] Alba C, Hufbauer RA (2012) Exploring the potential for climatic factors, herbivory, and co-occurring vegetation to shape performance in native and introduced populations of *Verbascum thapsus* . Biol Invasions 14: 2505–2518.

[30] Wilbur H, Hufbauer RA, Alba C, Norton A (2013) The effect of insect herbivory on the growth and fitness of introduced *Verbascum thapsus* L. Neobiota. 19: 21–44.

[31] Gross KL, Werner PA (1978) Biology of Canadian Weeds 28. *Verbascum thapsus* and *Verbascum blattaria* . Can J Plant Sci 58: 401–413.

[32] Mitich LW (1989) Common mullein: The roadside torch parade. Weed Technology 3: 704–705.

[33] Clapham AR, Tutin TG, Warburg EF (1952) *Flora of the British Isles*. University Press, Cambridge, UK.

[34] Alba C, Prioreschi R, Quintero C (2013) Population and leaf-level variation of iridoid glycosides in the invasive weed *Verbascum thapsus* L. (common mullein): implications for herbivory by generalist insects. Chemoecology 23: 83–92.

[35] Salisbury EJ (1942) *The Reproductive Capacity of Plants*. Bell, London.

[36] Bowers MD (1991) The iridoid glycosides. In: Rosenthal GA, Berenbaum MR, editors. Herbivores: Their Interactions with Secondary Plant Metabolites. Academic Press, New York.

[37] Dobler S, Petschenka G, Pankoke H (2011) Coping with toxic plant compounds—The insect's perspective on iridoid glycosides and cardenolides. Phytochemistry 72: 1593–1604. 21620425 10.1016/j.phytochem.2011.04.015

[38] Bowers MD, Puttick GM (1988) Response of generalist and specialist insects to qualitative allelochemical variation. J Chem Ecol 14: 319–334. 24277012 10.1007/BF01022549

[39] Bowers MD (1984) Iridoid glycosides and host-plant specificity in larvae of the buckeye butterfly, *Junonia coenia* (Nymphalidae). J Chem Ecol 10: 1567–1577. 24318391 10.1007/BF00988425

[40] Aranwela N, Sanson G, Read J (1999) Methods of assessing leaf-fracture properties. New Phytol 144: 369–383.

[41] Lucas PW, Turner IM, Dominy NJ, Yamashita N (2000) Mechanical defences to herbivory. Ann Bot 86: 913–920.

[42] Choong M (1996) What makes a leaf tough and how this affects the pattern of *Castanopsis fissa* leaf consumption by caterpillars. Funct Ecol 10: 668–674.

[43] Feeny P (1970) Seasonal changes in oak leaf tannins and nutrients as a cause of spring feeding by winter moth caterpillars. Ecology 51: 565–581.

[44] Clissold FJ, Sanson GD, Read J, Simpson SJ (2009) Gross vs. net income: How plant toughness affects performance of an insect herbivore. Ecology 90: 3393–3405. 20120808 10.1890/09-0130.1

[45] Robinson GS, Ackery PR, Kitching IJ, Beccaloni GW, Hernández LM (2010) HOSTS - A Database of the World's Lepidopteran Hostplants. Natural History Museum, London. Available: http://www.nhm.ac.uk/hosts. Accessed 2014 February 22.

[46] Jamieson MA, Bowers MD (2010) Iridoid glycoside variation in the invasive plant Dalmatian toadflax, *Linaria dalmatica* (Plantaginaceae), and sequestration by the biological control agent, *Calophasia lunula* . J Chem Ecol 36: 70–79. 20077129 10.1007/s10886-009-9728-z

[47] Sanson G, Read J, Aranwela N, Clissold F, Peeters P (2001) Measurement of leaf biomechanical properties in studies of herbivory: Opportunities, problems and procedures. Austral Ecol 26: 535–546.

[48] Hanson FE (1983) The behavioral and neurophysiological basis of food plant selection by lepidopterous larvae. In: Ahmad S, editor. Herbivorous Insects: Host-Seeking Behavior and Mechanisms. Academic Press, New York.

[49] Littell RC, Milliken GA, Stroup WW, Wolfinger RD (1996) SAS System for Mixed Models. SAS Institute, Cary, NC.

[50] Marak HB, Biere A, Van Damme JMM (2000) Direct and correlated responses to selection on iridoid glycosides in *Plantago lanceolata* L. J Evol Biol 13: 985–996.

[51] Agren J, Schemske DW (1994) Evolution of trichome number in a naturalized population of *Brassica rapa* . Am Nat 143: 1–13.

[52] Elle E, van Dam NM, Hare JD (1999) Cost of glandular trichomes, a "resistance" character in *Datura wrightii* Regel (Solanaceae). Evolution 53: 22–35. 28565189 10.1111/j.1558-5646.1999.tb05330.x

[53] van Dam NM, Hare JD, Elle E (1999) Inheritance and distribution of trichome phenotypes in *Datura wrightii* . J Hered 90: 220–227.

[54] Hemming JDC, Lindroth RL (1995) Intraspecific variation in aspen phytochemistry: Effects on performance of gypsy moths and forest tent caterpillars. Oecologia 103: 79–88. 28306948 10.1007/BF00328428

[55] Damtoft S (1994) Biosynthesis of catalpol. Phytochemistry 35: 1187–1189.

[56] Adler LS, Schmitt J, Bowers MD (1995) Genetic variation in defensive chemistry in *Plantago lanceolata* (Plantaginaceae) and its effect on the specialist herbivore *Junonia coenia* (Nymphalidae). Oecologia 101: 75–85. 28306979 10.1007/BF00328903

[57] Valkama E, Salminen JP, Koricheva J, Pihlaja K (2004) Changes in leaf trichomes and epicuticular flavonoids during leaf development in three birch taxa. Ann Bot 94: 233–242. 15238348 10.1093/aob/mch131 PMC4242156

[58] Valverde PL, Fornoni J, Nunez-Farfan J (2001) Defensive role of leaf trichomes in resistance to herbivorous insects in *Datura stramonium* . J Evol Biol 14: 424–432.

[59] Levin DA (1973) Role of trichomes in plant defense. Q Rev Biol 48: 3–15.

[60] Agrawal AA (1999) Induced responses to herbivory in wild radish: Effects on several herbivores and plant fitness. Ecology 80: 1713–1723.

[61] Chu C-C, Freeman TP, Buckner JS, Henneberry TJ, Nelson, et al (2001) Susceptibility of upland cotton cultivars to *Bemisia tabaci* biotype B (Homoptera: Aleyrodidae) in relation to leaf age and trichome density. Ann Entomol Soc Am 94: 743–749.

[62] Facknath S (2005) Leaf age and life history variables of a leafminer: The case of *Liriomyza trifolii* on potato leaves. Entomol Exp Appl 115: 79–87.

[63] Lowman MD, Box JD (1983) Variation in leaf toughness and phenolic content among 5 species of Australian rain-forest trees. Austr J Ecol 8: 17–25.

[64] Aide TM, Londono EC (1989) The effects of rapid leaf expansion on the growth and survivorship of a lepidopteran herbivore. Oikos 55: 66–70.

[65] Nichols-Orians CM, Schultz JC (1989) Leaf toughness affects leaf harvesting by the leaf cutter ant, *Atta cephalotes* (L) (Hymenoptera, Formicidae). Biotropica 21: 80–83.

[66] Wheeler GS (1996) Center TD (1996) The influence of *Hydrilla* leaf quality on larval growth and development of the biological control agent *Hydrellia pakistanae* (Diptera: Ephydridae). Biological Control 7: 1–9.

[67] Larsson S, Ohmart CP (1988) Leaf age and larval performance of the leaf beetle *Paropsis atomaria* . Ecol Entomol 13: 19–24.

[68] Alba C, Bowers MD, Blumenthal D, Hufbauer R (2011) Evolution of growth but not structural or chemical defense in *Verbascum thapsus* (common mullein) following introduction to North America. Biol Invasions 13: 2379–2389.

[69] Herms DA, Mattson WJ (1992) The dilemma of plants: to grow or defend. Q Rev Biol 67: 478–478.

[70] Koricheva J (2002) The carbon-nutrient balance hypothesis is dead; long live the carbon-nutrient balance hypothesis? Oikos 98: 537–539.

[71] Puttick GM, Bowers MD (1988) Effect of qualitative and quantitative variation in allelochemicals on a generalist insect: Iridoid glycosides and the southern army worm. J Chem Ecol 14: 335–351. 24277013 10.1007/BF01022550

