# Supplementary material for: Chemical and Mechanical Defenses Vary among Maternal Lines and Leaf Ages in Verbascum thapsus L. (Scrophulariaceae) and Reduce Palatability to a Generalist Insect
Source: PLoS One. 2014 Aug 15;9(8):e104889. doi: 10.1371/journal.pone.0104889 (PMC4134248; doi:10.1371/journal.pone.0104889)
Supplement: Table S3 — Raw data for feeding trials to determine the preference of Trichoplusia ni for young and old leaves of Verbascum thapsus . (DOC) [file pone.0104889.s003.doc]

Table S3. Raw data for feeding trials to determine the preference of *Trichoplusia ni* for young and old leaves of *Verbascum thapsus*.

| Maternal Line | Block | Leaf Age | Initial Area- Final Area | sqrt_Inititial-Final | Total area | Initial-Final/Initial | x100 |
| --- | --- | --- | --- | --- | --- | --- | --- |
| 1 | 1 | y | 0.035 | 0.187 | 76.373 | 0.000 | 0.046 |
| 1 | 1 | old | 8.351 | 2.890 | 106.347 | 0.079 | 7.852 |
| 4 | 1 | y | 0.000 | 0.000 | 102.218 | 0.000 | 0.000 |
| 4 | 1 | y | 0.023 | 0.153 | 94.940 | 0.000 | 0.025 |
| 4 | 1 | old | 3.156 | 1.776 | 100.894 | 0.031 | 3.128 |
| 4 | 1 | old | 6.534 | 2.556 | 100.430 | 0.065 | 6.506 |
| 5 | 1 | y | 2.574 | 1.604 | 123.037 | 0.021 | 2.092 |
| 5 | 1 | y | 0.000 | 0.000 | 63.409 | 0.000 | 0.000 |
| 5 | 1 | old | 1.053 | 1.026 | 100.022 | 0.011 | 1.052 |
| 5 | 1 | old | 9.301 | 3.050 | 52.487 | 0.177 | 17.720 |
| 6 | 1 | y | 0.000 | 0.000 | 82.720 | 0.000 | 0.000 |
| 6 | 1 | old | 4.487 | 2.118 | 90.960 | 0.049 | 4.933 |
| 7 | 1 | y | 0.212 | 0.460 | 76.974 | 0.003 | 0.275 |
| 7 | 1 | old | 0.352 | 0.593 | 95.581 | 0.004 | 0.368 |
| 8 | 1 | y | 0.359 | 0.599 | 92.867 | 0.004 | 0.386 |
| 8 | 1 | y | 0.197 | 0.443 | 87.842 | 0.002 | 0.224 |
| 8 | 1 | old | 3.315 | 1.821 | 108.698 | 0.030 | 3.049 |
| 8 | 1 | old | 5.654 | 2.378 | 104.173 | 0.054 | 5.427 |
| 9 | 1 | y | 0.363 | 0.603 | 91.843 | 0.004 | 0.396 |
| 9 | 1 | old | 2.334 | 1.528 | 94.563 | 0.025 | 2.468 |
| 1 | 2 | y | 0.064 | 0.254 | 19.772 | 0.003 | 0.325 |
| 1 | 2 | old | 1.127 | 1.062 | 32.744 | 0.034 | 3.442 |
| 2 | 2 | y | 9.399 | 3.066 | 39.764 | 0.236 | 23.637 |
| 2 | 2 | old | 0.051 | 0.226 | 60.034 | 0.001 | 0.085 |
| 3 | 2 | y | 10.232 | 3.199 | 45.855 | 0.223 | 22.314 |
| 3 | 2 | old | 0.497 | 0.705 | 42.296 | 0.012 | 1.175 |
| 4 | 2 | y | 0.056 | 0.236 | 43.109 | 0.001 | 0.129 |
| 4 | 2 | old | 8.144 | 2.854 | 43.985 | 0.185 | 18.514 |
| 5 | 2 | y | 1.043 | 1.021 | 26.742 | 0.039 | 3.902 |
| 5 | 2 | old | 3.844 | 1.961 | 40.805 | 0.094 | 9.421 |
| 6 | 2 | y | 0.346 | 0.588 | 27.531 | 0.013 | 1.255 |
| 6 | 2 | old | 1.802 | 1.343 | 50.018 | 0.036 | 3.604 |
| 7 | 2 | y | 0.142 | 0.376 | 27.723 | 0.005 | 0.511 |
| 7 | 2 | old | 2.275 | 1.508 | 43.457 | 0.052 | 5.236 |
| 8 | 2 | y | 0.269 | 0.518 | 40.772 | 0.007 | 0.659 |
| 8 | 2 | old | 1.020 | 1.010 | 56.466 | 0.018 | 1.807 |
| 9 | 2 | y | 1.143 | 1.069 | 30.018 | 0.038 | 3.809 |
| 9 | 2 | old | 1.549 | 1.245 | 47.172 | 0.033 | 3.285 |
| 10 | 2 | y | 0.671 | 0.819 | 13.575 | 0.049 | 4.940 |
| 10 | 2 | old | 6.138 | 2.477 | 24.509 | 0.250 | 25.044 |
