# Supplementary material for: Chemical and Mechanical Defenses Vary among Maternal Lines and Leaf Ages in Verbascum thapsus L. (Scrophulariaceae) and Reduce Palatability to a Generalist Insect
Source: PLoS One. 2014 Aug 15;9(8):e104889. doi: 10.1371/journal.pone.0104889 (PMC4134248; doi:10.1371/journal.pone.0104889)
Supplement: Table S4 — Raw data for feeding trials to determine the preference of Trichoplusia ni for Verbascum thapsus leaves with and without trichomes. (DOC) [file pone.0104889.s004.doc]

Table S4. Raw data for feeding trials to determine the preference of *Tricho-*

*plusia ni* for *Verbascum thapsus* leaves with and without trichomes.

| Maternal Line | Treatment | Initial Area-Final Area | Total Area | Initial-Final/Initial | x100 |
| --- | --- | --- | --- | --- | --- |
| 1 | Unshaved | 0.5706 | 12.3737 | 0.0461 | 4.6114 |
| 2 | Unshaved | 0.3054 | 19.2024 | 0.0159 | 1.5904 |
| 3 | Unshaved | 0 | 23.7141 | 0.0000 | 0.0000 |
| 4 | Unshaved | 0.6734 | 16.7287 | 0.0403 | 4.0254 |
| 5 | Unshaved | 0.8319 | 20.6292 | 0.0403 | 4.0326 |
| 5 | Unshaved | 0.1232 | 12.9635 | 0.0095 | 0.9504 |
| 6 | Unshaved | 0.0378 | 18.2568 | 0.0021 | 0.2070 |
| 7 | Unshaved | 0.7363 | 16.4412 | 0.0448 | 4.4784 |
| 8 | Unshaved | 0.4733 | 20.8291 | 0.0227 | 2.2723 |
| 9 | Unshaved | 0 | 15.2999 | 0.0000 | 0.0000 |
| 10 | Unshaved | 0.2596 | 12.3162 | 0.0211 | 2.1078 |
| 1 | Shaved | 4.5588 | 11.8794 | 0.3838 | 38.3757 |
| 2 | Shaved | 2.348 | 13.8483 | 0.1696 | 16.9551 |
| 3 | Shaved | 5.1666 | 21.7499 | 0.2375 | 23.7546 |
| 4 | Shaved | 3.8145 | 16.0793 | 0.2372 | 23.7230 |
| 5 | Shaved | 4.6233 | 19.8957 | 0.2324 | 23.2377 |
| 5 | Shaved | 1.6048 | 11.8461 | 0.1355 | 13.5471 |
| 6 | Shaved | 2.9911 | 17.359 | 0.1723 | 17.2308 |
| 7 | Shaved | 2.1737 | 15.2444 | 0.1426 | 14.2590 |
| 8 | Shaved | 3.6309 | 20.7674 | 0.1748 | 17.4837 |
| 9 | Shaved | 2.3672 | 15.8133 | 0.1497 | 14.9697 |
| 10 | Shaved | 1.0034 | 11.356 | 0.0884 | 8.8359 |
